# Strong Isotope Effects on Melting Dynamics and Ice Crystallisation Processes in Cryo Vitrification Solutions

**DOI:** 10.1371/journal.pone.0120611

**Published:** 2015-03-27

**Authors:** Oleg Kirichek, Alan Soper, Boris Dzyuba, Sam Callear, Barry Fuller

**Affiliations:** 1 ISIS facility, STFC, Rutherford Appleton Laboratory, Harwell Oxford Campus, Didcot, Oxon, United Kingdom; 2 South Bohemian Research Center of Aquaculture and Hydrocenoses, University of South Bohemia in Ceske Budejovice, Zatisi, Vodnany, Czech Republic; 3 Department of Surgery & Liver Transplant Unit, University College London, UCL Royal Free Campus, London, United Kingdom; Martin-Luther-Universität Halle-Wittenberg, GERMANY

## Abstract

The nucleation and growth of crystalline ice during cooling, and further crystallization processes during re-warming are considered to be key processes determining the success of low temperature storage of biological objects, as used in medical, agricultural and nature conservation applications. To avoid these problems a method, termed vitrification, is being developed to inhibit ice formation by use of high concentration of cryoprotectants and ultra-rapid cooling, but this is only successful across a limited number of biological objects and in small volume applications. This study explores physical processes of ice crystal formation in a model cryoprotective solution used previously in trials on vitrification of complex biological systems, to improve our understanding of the process and identify limiting biophysical factors. Here we present results of neutron scattering experiments which show that even if ice crystal formation has been suppressed during quench cooling, the water molecules, mobilised during warming, can crystallise as detectable ice. The crystallisation happens right after melting of the glass phase formed during quench cooling, whilst the sample is still transiting deep cryogenic temperatures. We also observe strong water isotope effects on ice crystallisation processes in the cryoprotectant mixture. In the neutron scattering experiment with a fully protiated water component, we observe ready crystallisation occurring just after the glass melting transition. On the contrary with a fully deuteriated water component, the process of crystallisation is either completely or substantially supressed. This behaviour might be explained by nuclear quantum effects in water. The strong isotope effect, observed here, may play an important role in development of new cryopreservation strategies.

## Introduction

The cryopreservation process whereby cells, tissue samples or embryos are preserved by cooling to sub-zero temperatures is broadly used in medicine, agriculture and nature conservation programs [1]. Modern cryopreservation technology that deals with small objects such as sperm or mammalian embryos provides efficient preservation under cryogenic conditions for many years. However for biological objects of larger volumes, or with a sensitivity to how they are cooled, this technology is limited by a risk of sample damage by the kinetics of the cooling profile and formation of intra and extra-cellular ice. In some cases this problem can be solved by using of vitrification—a cooling method that leads to the solidification of water without formation of ice crystals. In fact vitrification offers promising outcomes in cases where other methods of freezing do not [2]. To achieve the vitrification a high concentration of cryoprotectants and rapid cooling rates are required [3]. This approach has the potential to be applied for vitrification of complex biological systems, for example fish oocytes and embryos, where successful cryopreservation has still not been achieved [4]. However a major challenge for successful recovery from vitrification is the potential injury which appears to be linked more strongly with the warming phase. Observational studies by cryo-microscopy [5, 6] have indicated that ice crystallisation may occur as the sample passes through glass transition range of the solute mixture, whilst still at relatively low sub-zero temperatures (in the region above about 190 K). The exact nature of the molecular biophysical events in this region, and their relationship with processes which take place during the cooling phase, remain to be fully explained.

Here we present the results of neutron scattering measurements which show that even if crystal formation has been prevented during quench cooling of the sample the water in it can crystallise during warming up. The crystallisation happens immediately after the melting of the glassy phase that formed during the initial quench cooling of the sample. We have also observed strong isotope effects on the ice crystallisation processes in cryoprotectant mixture. For the fully protiated sample we observe clear crystallisation that occurs just after the glass melting transition (around 150 K). On the contrary, in the case of the fully deuteriated sample, the process of crystallisation is either completely or substantially suppressed. This behaviour might be explained by nuclear quantum effects in water and can have a significant impact on development of new vitrification technologies.

## Experimental Methods and Results

The neutron scattering data were obtained on the small angle diffractometer SANDALS [8] at the ISIS Facility, STFC Rutherford Appleton Laboratory, UK. In the experiment we studied cryo-protectant solutions sample with 23vol% 1,2-propanediol (PD), 17vol% methanol (Met), 20vol% dimethylsulphoxide (DMSO) mixture in water, the sample was formulated during a previous study of fish (Common carp) embryo cryopreservation [7]. The required chemical components were obtained from Sigma-Aldrich and mixed by volume to the required concentrations. The pure cryopreservation solutions were studied on their own, that is without added biological material. In the vitrification experiments, we used both deuteriated and protiated samples. Two cubic centimetres of the sample solution were sealed inside the experimental cell made of TiZr “zero” alloy. The sample and container were quench-cooled to 77 K by immersing in liquid nitrogen. The time required to reach thermal equilibrium was less than 40 seconds as detected by the boiling phase of the liquid nitrogen. After keeping the sample in the liquid nitrogen for another minute, the cell was quickly withdrawn and loaded into the variable temperature insert of a top-loading cryogen free cryostat, based on a pulse tube refrigerator [9], which was already installed on the neutron beam line. In order to prevent condensation of air and water vapour during the loading of the sample into the cryostat, the helium gas was purged through the insert and the insert heat-exchanger temperature was controlled at ∼100 K. Once the sample container was installed the insert was evacuated and a small portion of helium gas was added back in order to provide a thermal link between the insert heat-exchanger and the sample cell. The temperature of the insert was quickly reduced to 80K and then controlled at this level until thermal equilibrium was achieved within half an hour. After the collection of neutron diffraction data at this temperature was completed the sample cell was warmed up by 20K, stabilised at the next temperature and neutron diffraction measured again. The temperature range of the experiment was from 80 K to 225 K. In some experimental runs the diffraction measurements have been continued, after reaching the highest temperature, by step cooling back to 80 K conditions. The neutron scattering background signal from the empty TiZr container, empty cryostat and empty instrument were subtracted from the experimental data, using separate measurements, and the data put on absolute scale of differential scattering cross section by comparison with scattering from a slab of vanadium.

**Fig 1:**
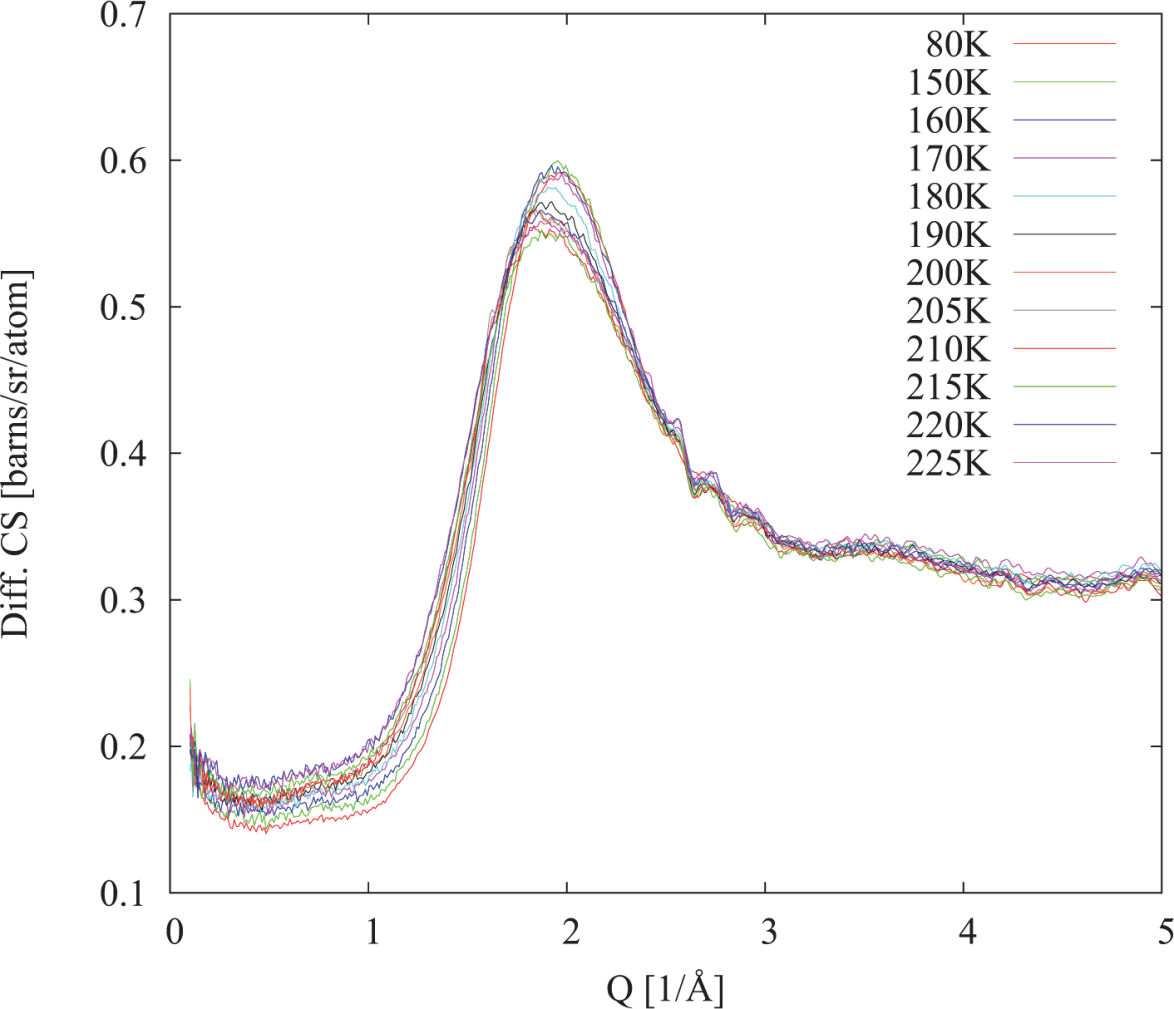
Differential cross sections for fully deuteriated sample. Heating after initial quench cooling.

**Fig 2:**
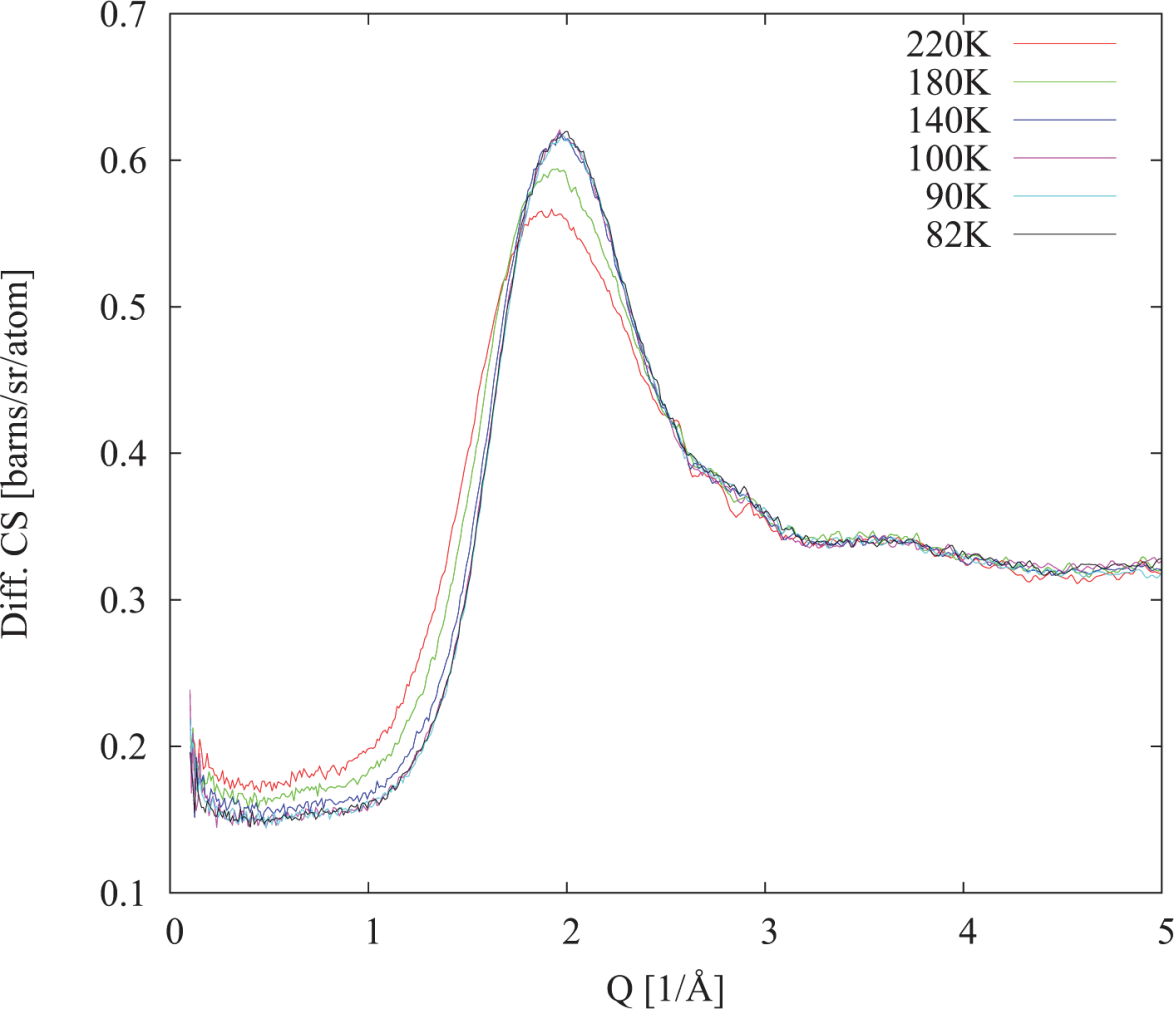
Differential cross sections for fully deuteriated sample. Cooling after first heating scan.

**Fig 3:**
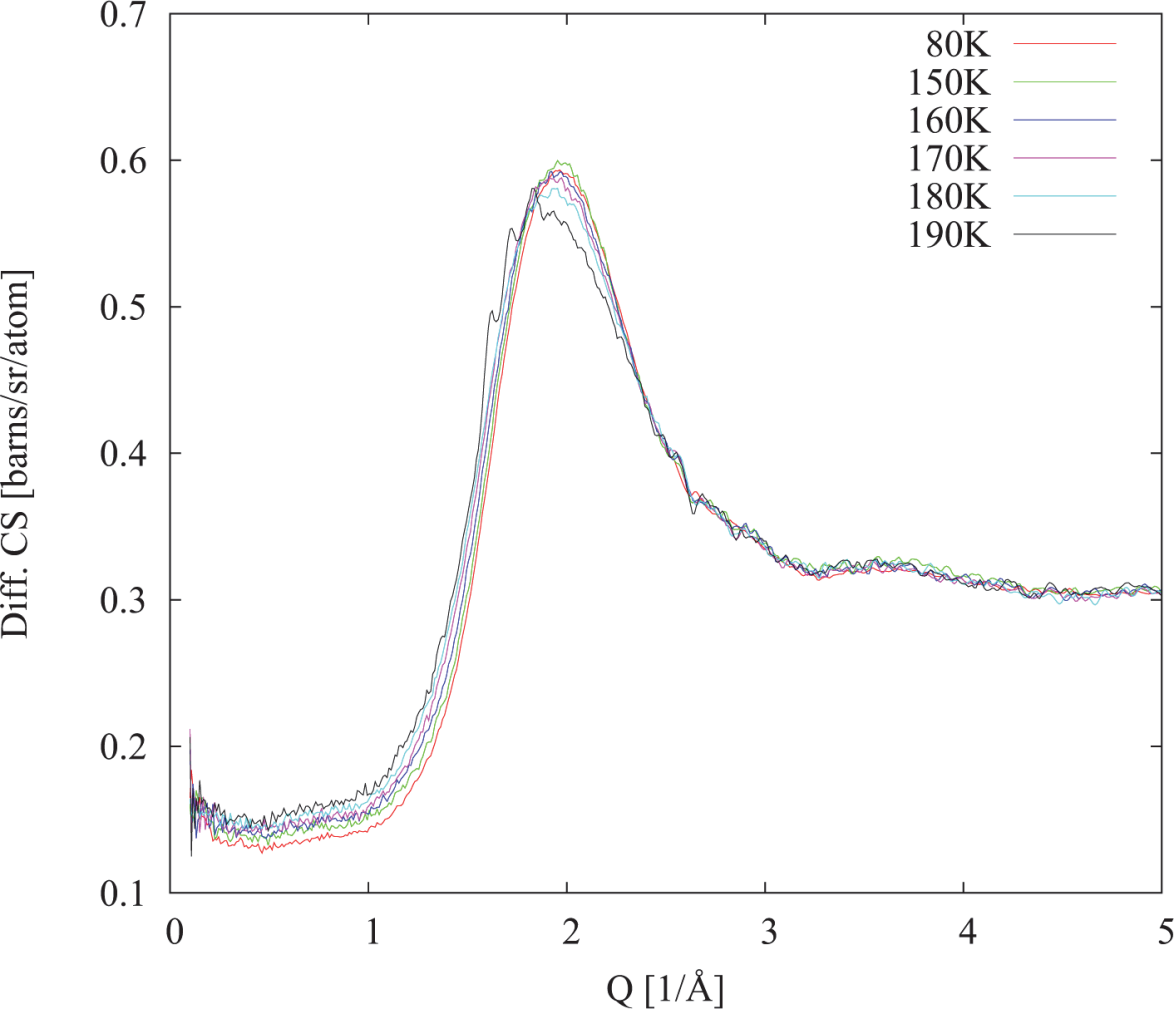
Differential cross sections for fully deuteriated sample. Second heating after initial quench cooling. The small peaks seen in the 190K data indicate some ice crystallisation in this sample (for more details see text).

As it is possible to see in Fig. 1 during heating up of fully deuteriated sample (40% D_2_O; 23% PD (d); 17% Met (d); 20% DMSO (d)) there was no sign of crystal formation across the entire temperature range. The slight changes in diffraction signal can be explained by melting of the glass phase that occurs around 155 K. Data collected during the cooling phase, started after completion of the first heating phase at temperature 225 K, are presented in Fig. 2. Similarly, as in the previous run, there was no indication of crystal formation during the second cooling. The sample remains liquid until 155 K where glass solidification transition took place. The second heating run presented in Fig. 3 has been performed with the same sample in order to check reproducibility of the results. As in the first run the glassy phase melted at 155 K. However this time at 190 K, weak crystallite diffraction peaks can be observed. These peaks disappear upon further heating.

**Fig 4:**
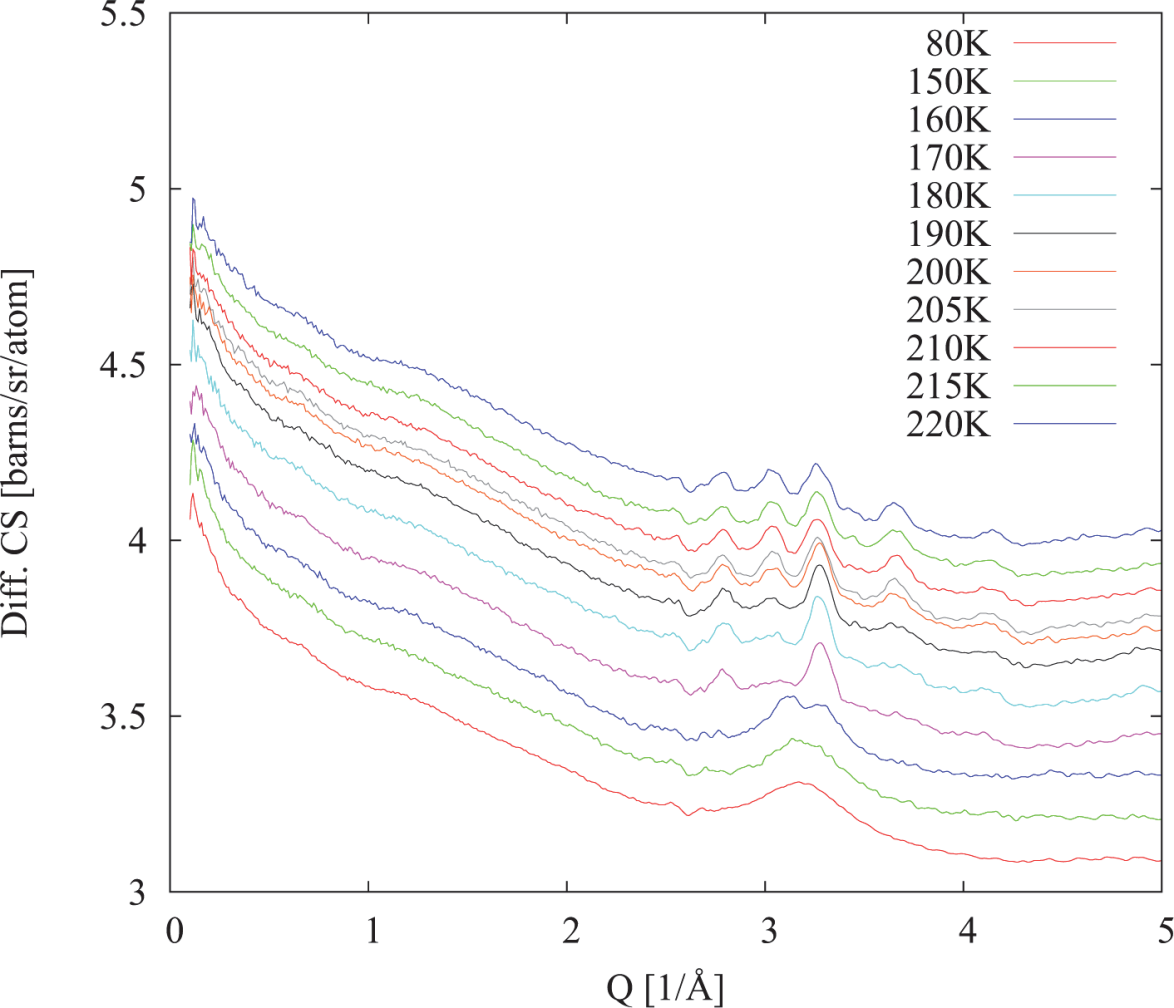
Differential cross sections for fully protiated sample. Heating after initial quench cooling. The crystallisation transition occurs between 160 K and 170 K.

**Fig 5:**
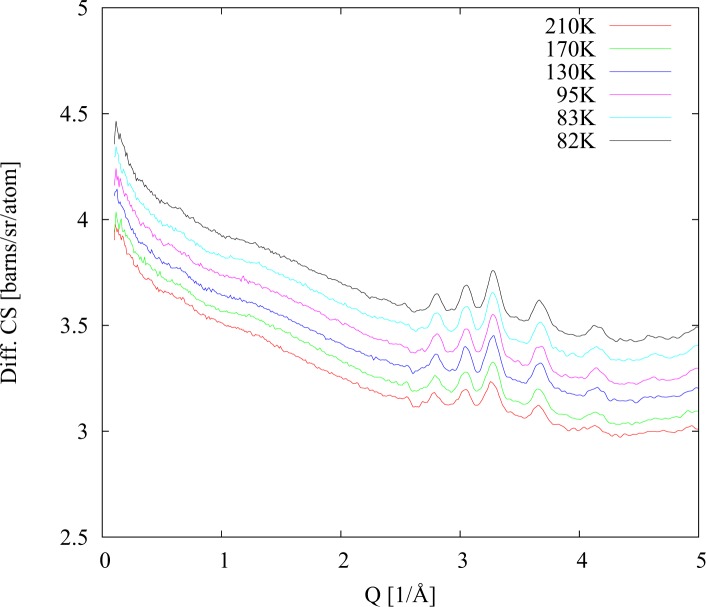
Differential cross sections for fully protiated sample. Cooling after heating scan.

The situation changed dramatically in case of the fully protiated sample (40% H_2_O; 23% PD (h); 17% Met (h); 20% DMSO (h)). As we can see in Fig. 4 the sample was again in the glassy state at temperatures below 150 K. However at 160 K, immediately above glass melting transition, the strong diffraction peaks from water crystallites appear in the data. Once they have appeared these peaks remain in the diffraction data during heating up to the maximum temperature 220 K, as well as during consequent second cooling run back to 82 K (see Fig. 5). The diffraction signal does not change even around the glass solidification transition at 150 K. (Note that due to the change in scattering length from +6.67fm for the deuteron compared to −3.74fm for the proton the Bragg peaks in the protiated sample occur at quite different Q values compared to the deuteriated sample. The different position of these peaks is a direct indication of ice Ih formation.).

From their positions, all the crystallite diffraction peaks correspond to pure water (or heavy water) ice Ih, and are not related to the solid phases of the other cryoprotectant mixture components which have other distinct peak assignments.

For our differential scanning calorimetry (DSC) measurements we have used a standard DSC Q2000 to determine the heat flow associated with phase transitions as a function of temperature. 10 mm^3^ of the sample mixture was sealed in aluminium hermetic pans. The sealed pan was initially quench-cooled to 77 K by immersing in liquid nitrogen. The time required to reach thermal equilibrium was less than 3 seconds. After that the sample pan was loaded into the DSC cell pre-filled with cold nitrogen gas in order to prevent atmospheric water condensation. Once the cell was closed nitrogen was replaced by helium gas and the systems remained at 100 K for ten minutes in order to reach equilibrium. All DSC data presented here have been obtained at scanning rate dT/dt = 5 K/min in temperature range from 100 K to 300 K, which was provided by a Q2000 liquid nitrogen cooling system. Characteristic signatures of melting or formation of glass or/and crystallites can be found in [10, 11].

**Fig 6:**
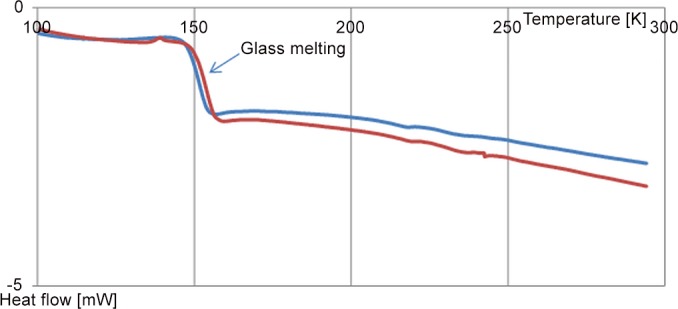
DSC temperature scan. Fully protiated sample—blue curve and fully deuteriated sample—red curve.

In Fig. 6 both fully protiated (40% H_2_O; 23% PD (h); 17% Met (h); 20% DMSO (h)) and fully deuteriated (40% D_2_O; 23% PD (d); 17% Met (d); 20% DMSO (d)) samples demonstrate a clear glass melting transition around 155 K (hydrogenated sample melts at a slightly colder temperature). These temperatures agree well with temperatures of glass melting transitions observed in the neutron diffraction measurements. Once melted the samples remain in liquid state on further warming up to 300 K. Weak features in the DSC trace between 200K—250K might be an indication of a small amount of ice formation.

## Discussion

As it follows from our neutron scattering experiments the process of water crystallization during warming from the vitrified state can be either significantly suppressed or completely avoided in the case of the deuteriated sample. The positions of the diffraction peaks in this case indicate that the crystallisation that occurs is due to ice formation—it does not involve crystallisation of the other components. In addition the degree of crystallinity in this sample is affected by the previous thermal history. On the other hand for the protiated sample crystallisation appear to involve all of the sample volume and is unaffected by previous thermal history. After crystallisation has happened this sample stays frozen across a broad temperature range.

The picture of crystallisation in the DSC samples differs from that observed in the neutron scattering experiment. We clearly observe a glass melting transition around 155K, but we do not see full crystallisation in the DSC deuteriated or protiated samples in the full-strength cryoprotectant mixture, although some very weak crystallisation may have occurred. This dissimilar behaviour can be explained by significant differences in the sample’s size: the DSC sample volume (10 μL) is ∼ 200 times smaller than that used in the neutron scattering experiments (2 mL) and the temperature scan time scale: DSC scan between 100K and 225K takes 25 min as in case of neutron scattering experiment the same temperature range is scanned in 17 hours. Another significant difference is the time taken to quench samples which is more than order of magnitude shorter for DSC sample in comparison with the neutron scattering data.

One of the most plausible explanations of the remarkably strong isotope effect observed in neutron scattering experiment could be a significant role of nuclear quantum effects in water. Recent quantum path integral simulations [12, 13] suggested that quantum effects weaken the hydrogen-bonding network in liquid water and make it less structured than its classical counterpart. The structural differences between heavy and light water at room temperature caused by nuclear quantum effects have been also observed experimentally [14, 15]. Even though nuclear quantum effects are significant their net effect on macroscopic properties in room temperature region is relatively small. For example the melting temperatures of light and heavy water differ by less than 4K. Recent experimental and theoretical results explain the situation by partial cancellation between quantum effects in the intra- and intermolecular components of the hydrogen bond [16]. However as it was shown in recent theoretical study at low temperatures the quantum effect such as tunnelling can significantly broaden the super-cooled liquid regime and affect glass formation process [17]. Moreover, recent simulations of Lennard-Jones system suggest that quantum effects might play a role in the dynamics of glass forming liquids and can either accelerate [18] or slow down [19] the dynamics. According to estimation in [17] the zero-point vibrational contribution to mean squared atomic displacement (which is known to play important role in the solid-liquid transition) in water at 136 K is 40% of the total value. Therefore the quantum fluctuations might significantly affect the dynamics of water at low temperatures. Finally, the zero point kinetic energy studied experimentally in a broad temperature range between 5K and 271K is also found to be approximately a factor ∼ 5 smaller than in the case of liquid water, highlighting the role played by anharmonic quantum fluctuations in the two phases [20]. The dramatic difference in crystallisation dynamics between deuteriated and protiated cryo-protectant solutions observed in our experiments highlights the role of nuclear quantum effects discussed above. Fundamentally, hydrogen is more delocalised than deuterium due to its higher zero point fluctuations, which can result in a higher diffusion coefficient and which can also speed up other mass and heat transport processes. That, in turn, accelerates the water crystal nucleation and growth.

## Conclusions

In conclusion, in our neutron scattering experiment we show that in cryoprotectant mixture samples, where ice crystal formation has been prevented by quench cooling, the water can crystallise during warming up. The crystallisation occurs soon after the glass melting transition (around 155 K). We also observed a strong isotope effect on crystallisation dynamics in the cryoprotectant mixture. In the neutron scattering experiments we observed clear ice crystallisation in the fully protiated samples. On the contrary in the case of the fully deuterated sample, the process of crystallisation was either significantly suppressed or absent. This behaviour might be explained by nuclear quantum effects in super-cooled water. The strong isotope effect, observed in our experiments, may play an important role in the development of new cryopreservation technologies.
